# Supplementary figures and images for: Fusogenic structural changes in arenavirus glycoproteins are associated with viroporin activity
Source: PLoS Pathog. 2023 Jul 26;19(7):e1011217. doi: 10.1371/journal.ppat.1011217 (PMC10406333; doi:10.1371/journal.ppat.1011217)

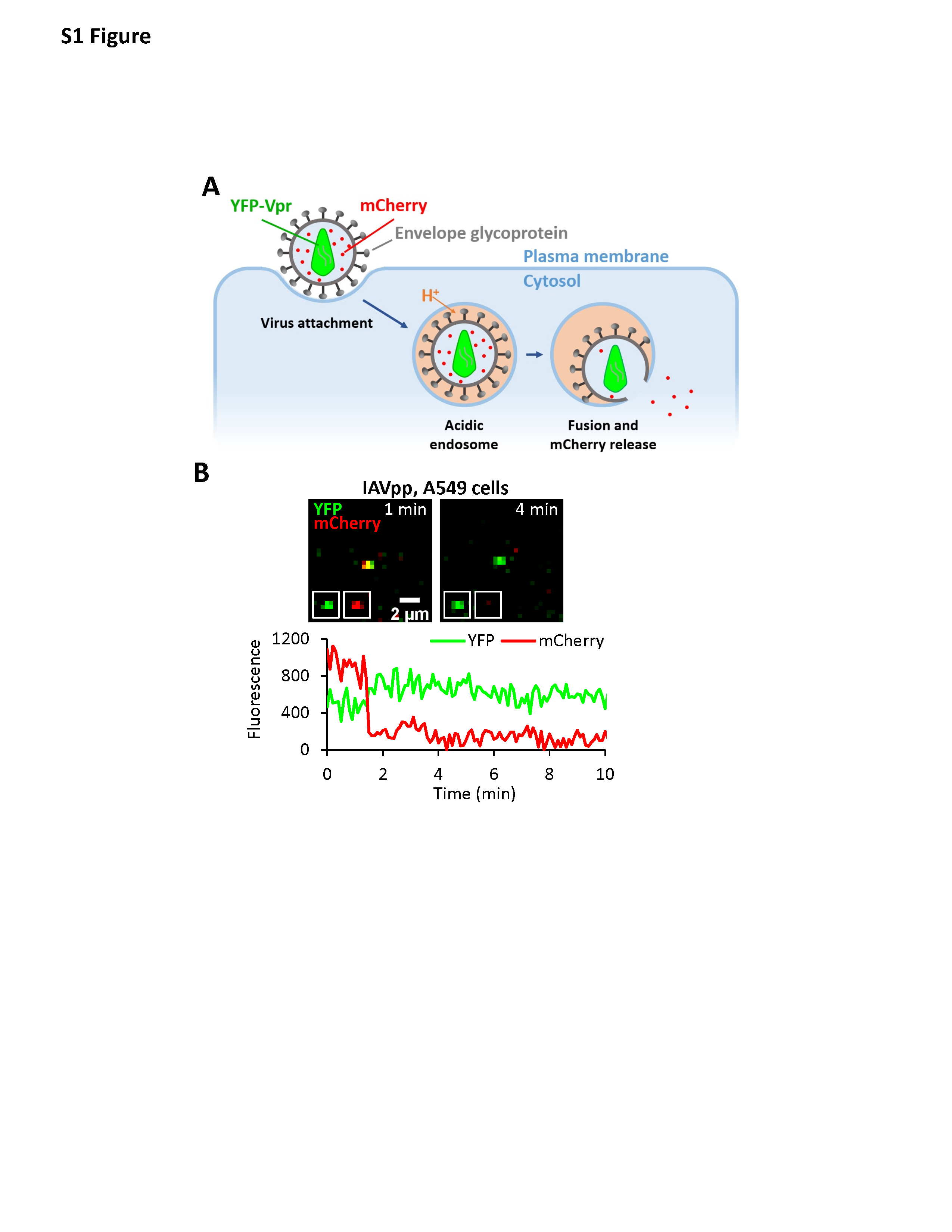

Supplement: S1 Fig — (A) Illustration of mCherry-CL-YFP-Vpr labeled single IAVpp fusion. IAVpp is internalized and trafficked to acidic endosomes where it fuses with the endosomal membrane without prior membrane permeabilization (YFP quenching). IAVpp-endosome fusion results in mCherry release into the cytoplasm. (B) A single IAVpp fusion event in A549 cell. Time-lapse images (top) and fluorescence traces (bottom) show virus fusion (mCherry loss) at 1.6 min (see S2 Movie). (TIF) [file ppat.1011217.s001.tif]

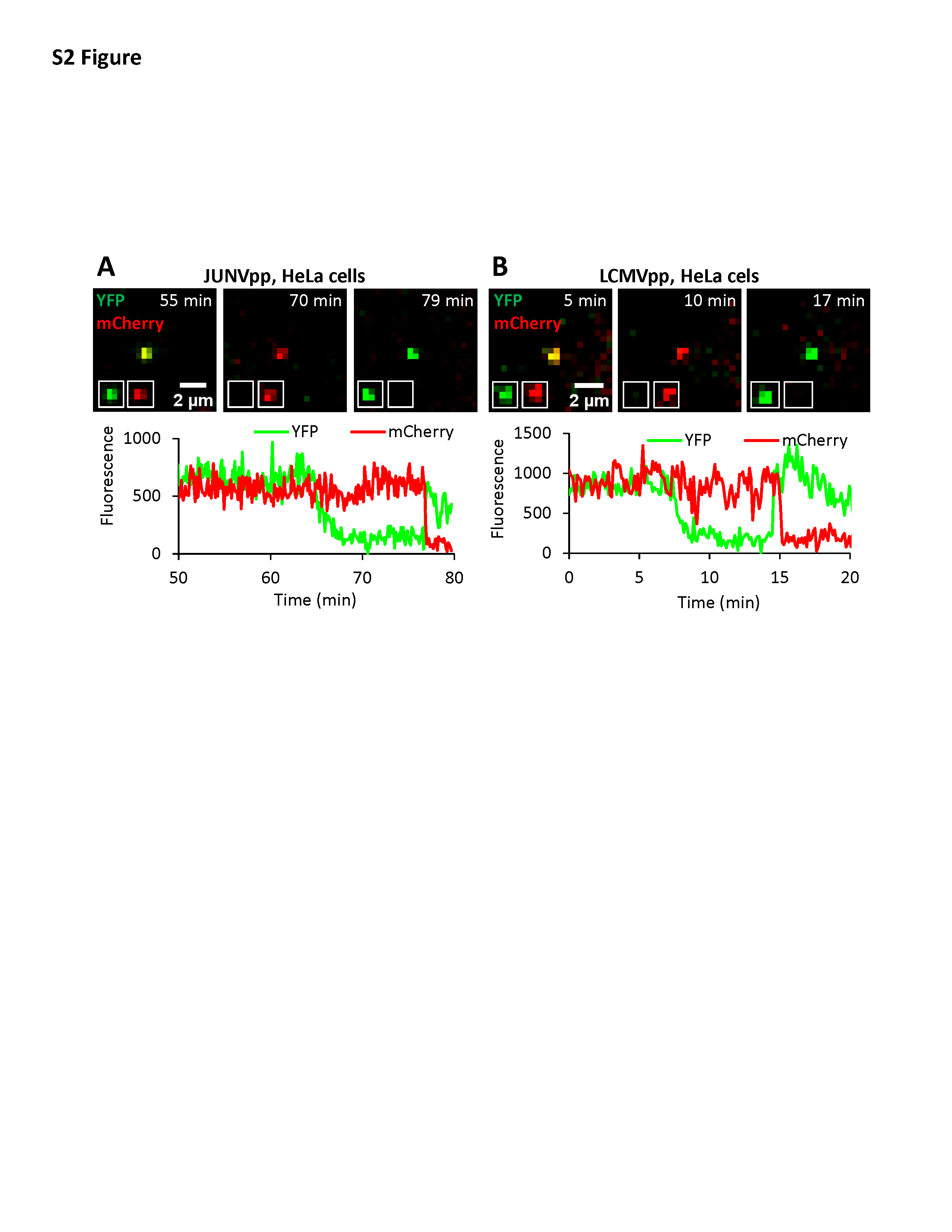

Supplement: S2 Fig — (A) A representative single JUNVpp fusion event in HeLa cell. Time-lapse images (top) and fluorescence traces (bottom) show YFP quenching at 68.0 min and fusion (YFP dequenching and mCherry loss) at 77.1 min. (B) A representative single LCMVpp fusion event in HeLa cell. Time-lapse images (top) and fluorescence traces (bottom) show YFP quenching at 8.8 min and fusion (YFP dequenching and mCherry loss) at 14.9 min. A slightly delayed mCherry release after YFP dequenching in A and C is due to a slower dilation of fusion pores to sizes that allow mCherry release. (TIF) [file ppat.1011217.s002.tif]

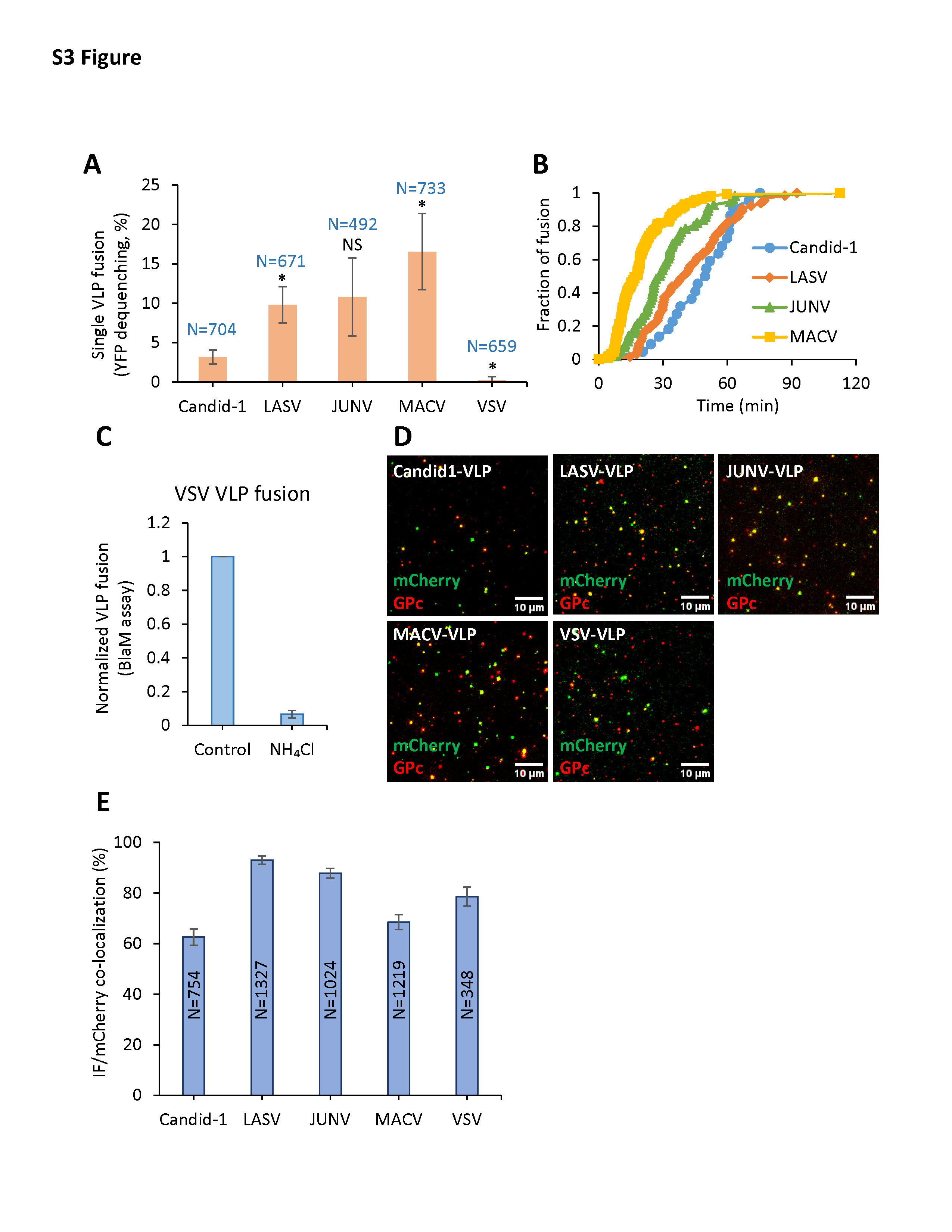

Supplement: S3 Fig — (A) Fusion efficiency (% of cell bound-particles that fused) of single Candid-1, LASV, JUNV, MACV and VSV VLPs in A549 cells measured by imaging. Data shown are means ± SD of 3 independent experiments. Results were analyzed by Student’s t-test. The number of total VLP particles analyzed is shown above the bars. Asterisks and NS on the top of bar represent the significance relative to the single Candid-1 VLP fusion efficiency in A549 cells. *, p<0.05; NS, not significant. (B) Kinetics of fusion of Candid-1, LASV, JUNV and MACV GPc VLPs. (C) VSV-G VLP fusion measured by BlaM assay. (D) Images of VLPs labeled with NP-DYFP/NP-DmCherry. VLPs were bound to poly-L-lysine coated coverslips, fixed, and incubated with antibodies against corresponding viral envelope proteins, followed by immunostaining with antibodies specific to the GPc panel. The mCherry marker of VLPs was used to identify VLP particles and visualize the associated envelope protein signal. (E) Quantification of co-localization of envelope protein immunofluorescence with viral particles identified by mCherry fluorescence. The numbers on the bars are the total numbers of VLP particles analyzed. Data shown are means ± SD of 4 imaging fields. (TIF) [file ppat.1011217.s003.tif]

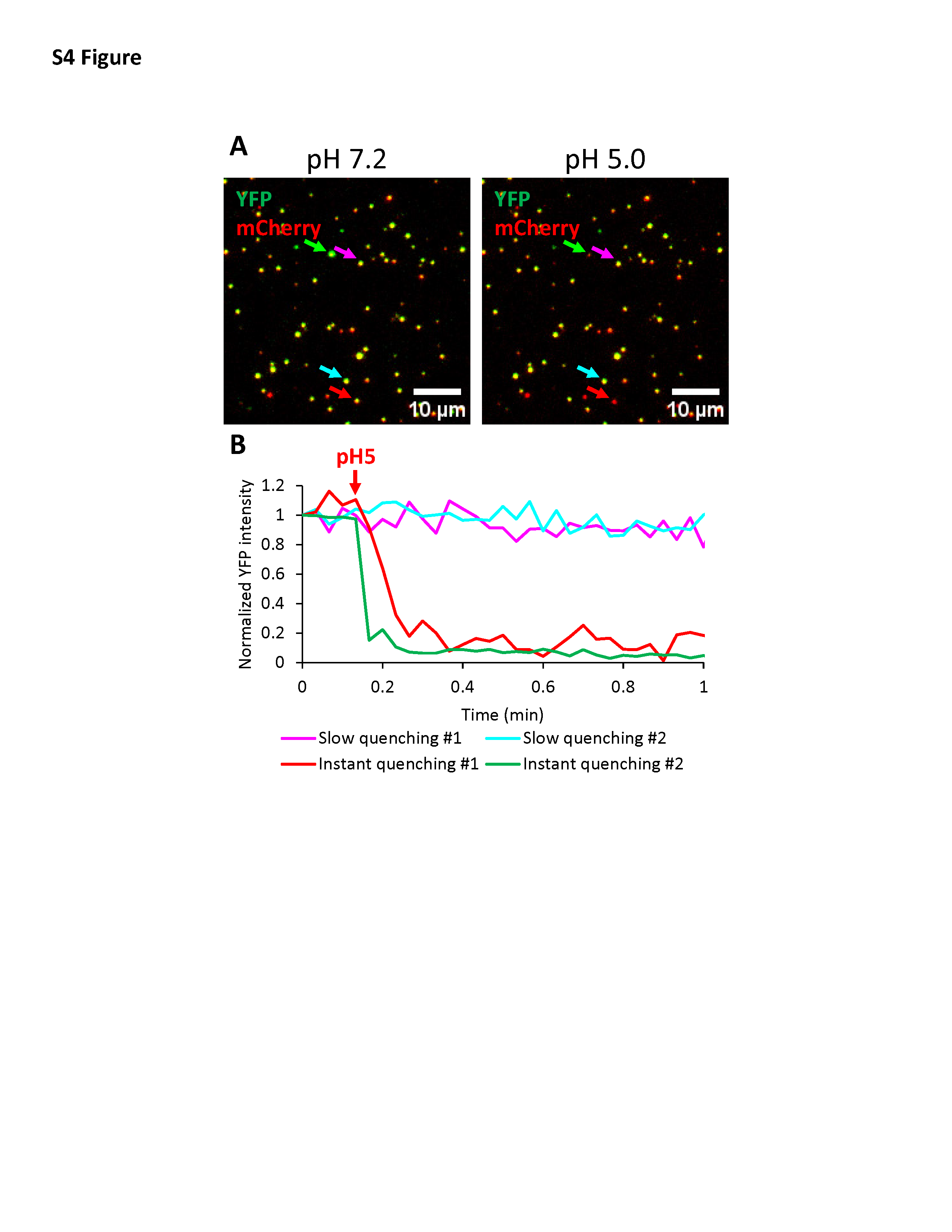

Supplement: S4 Fig — (A) Images of coverslip-adhered LASVpp in PBS (left) and 16 seconds after applying membrane-impermeable citrate pH 5.0 buffer (right). (B) Normalized YFP intensity of the only two particles that undergo instant YFP quenching and two representative particles that limit proton diffusion across their membranes. The point of adding a low pH buffer is marked with a red arrow. Tracked particles in (B) are marked by color-matched arrows in (A). Instant YPF quenching events constitute 3.9% of all particles. As shown in Fig 4, the YFP signal from the rest of single pseudovirions gradually decays over the course of several minutes (not noticeable on a short time scale shown in panel B), due to a baseline proton diffusion or due to increased membrane permeability associated with GPc refolding. (TIF) [file ppat.1011217.s004.tif]

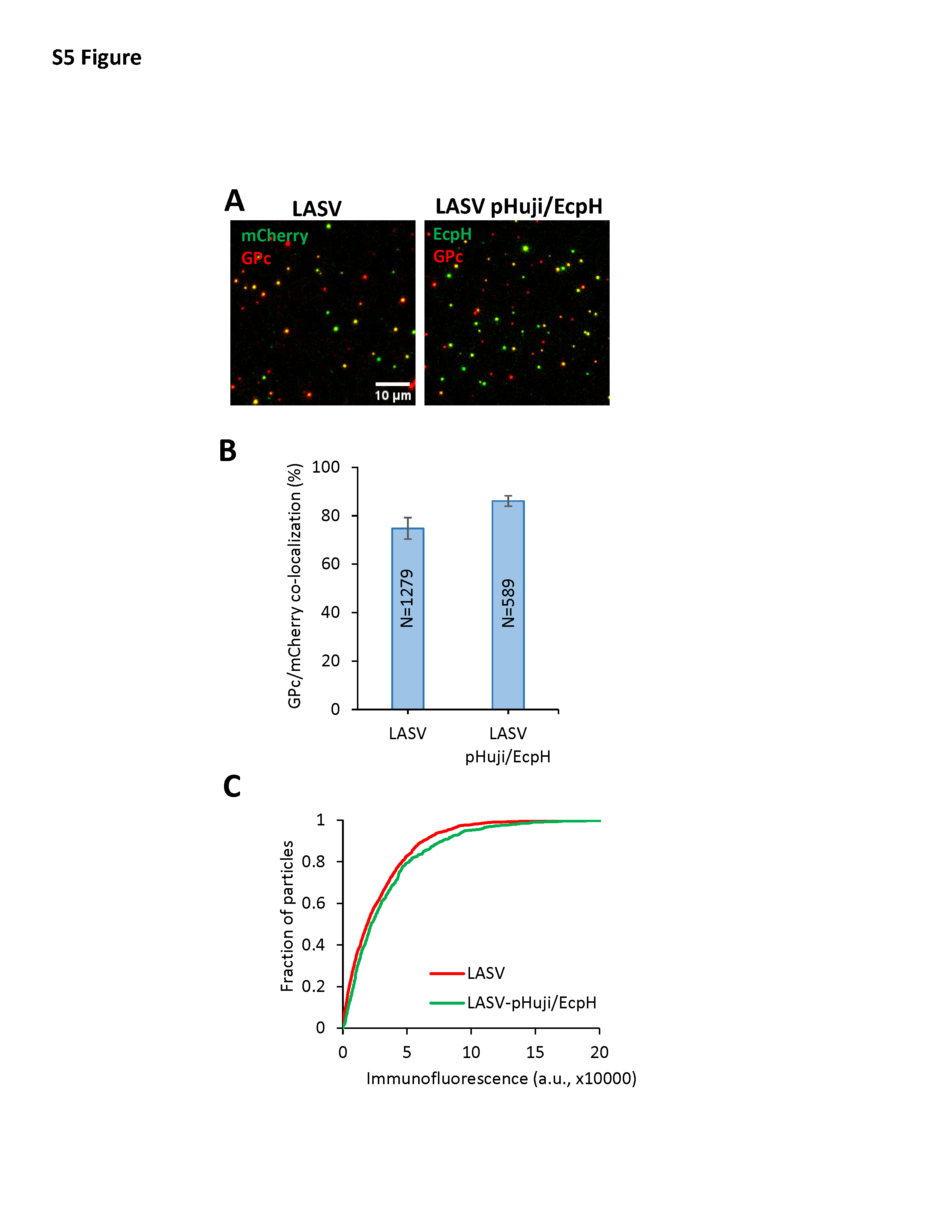

Supplement: S5 Fig — (A) Images of LASVpp labeled with mCherry-YFP-Vpr or pHuji/Gag-EcpH. Pseudoviruses were bound to poly-L-lysine coated coverslips, fixed, and incubated with anti-LASV GPc human antibody, followed by staining with anti-human AF647-conjugated antibody. Viral mCherry or EcpH markers were used to identify viral particles and visualize the associated GPc signal. (B) Quantification of co-localization of LASV GPc immunofluorescence with viral particles identified by mCherry or EcpH fluorescence, as indicated. The numbers of LASVpp analyzed are shown above the bars. Data shown are means ± SD of 4 imaging fields. (C) Cumulative distributions of the GPc immunofluorescence intensities for each virus preparation. (TIF) [file ppat.1011217.s005.tif]

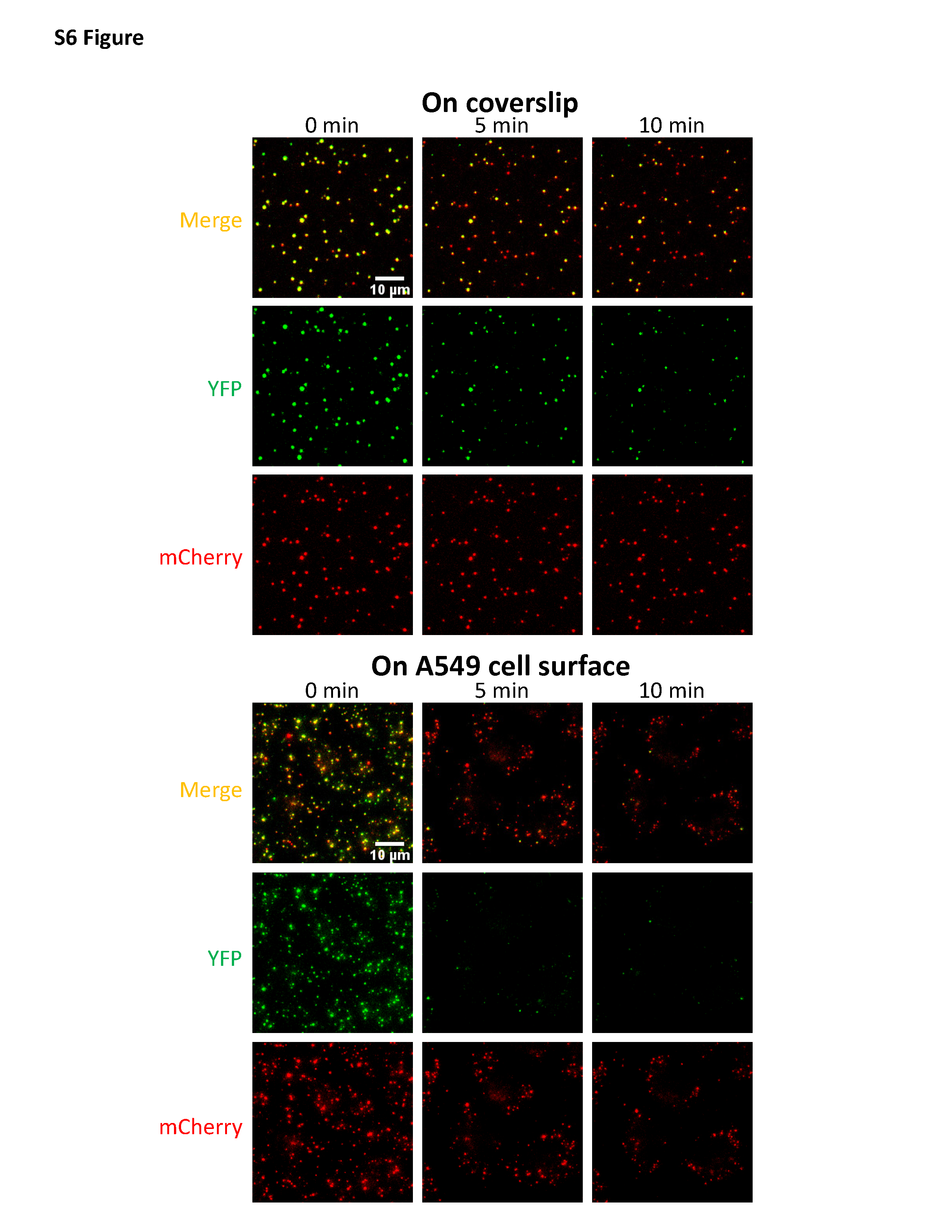

Supplement: S6 Fig — YFP fluorescence quenching at low pH is readily detectable, while the reference mCherry signal is largely unchanged. The apparent loss of mCherry puncta on A549 cells (lower panel) is due to cell shrinkage at low pH which results in a fraction of particles moving out of focus. (TIF) [file ppat.1011217.s006.tif]

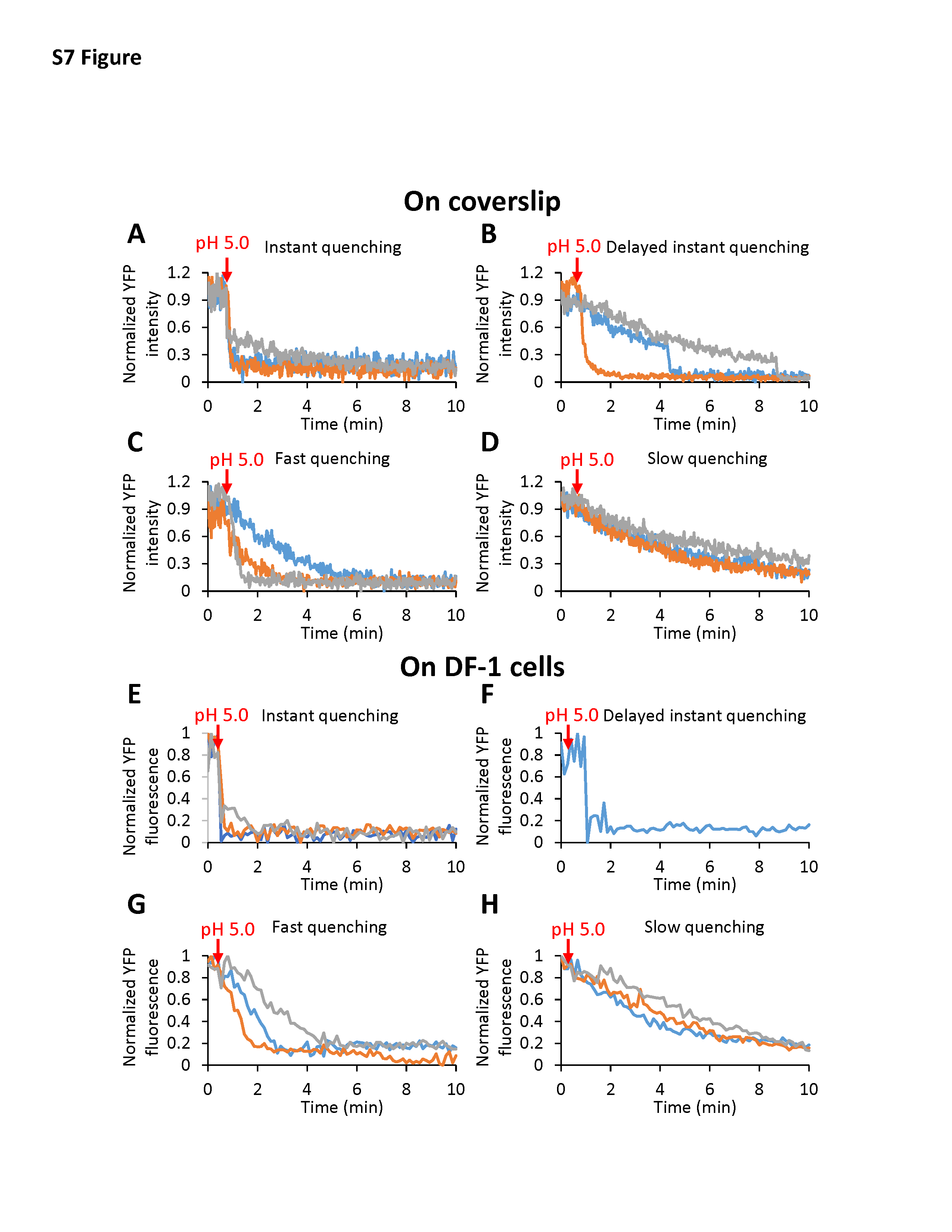

Supplement: S7 Fig — The time points when low pH buffer was added are marked with red arrows. See Fig 4. (TIF) [file ppat.1011217.s007.tif]

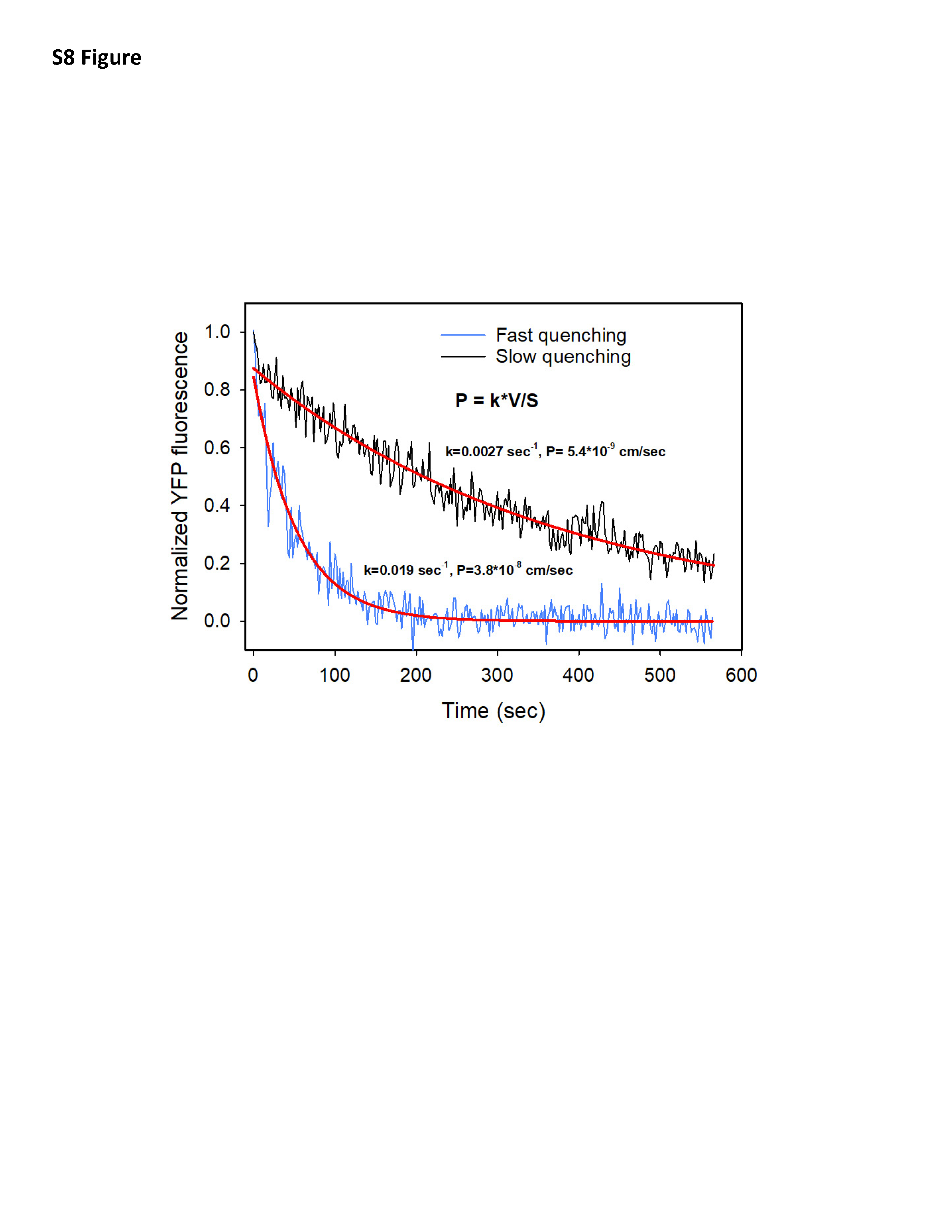

Supplement: S8 Fig — Representative pseudoviruses exhibiting fast and slow YFP quenching were used to estimate their membrane permeability to protons using the equation P = k*V/S, where k is the permeability coefficient determined by exponential fit of normalized YFP fluorescence decay (assuming that fluorescence changes reflect changes in intraviral proton concentration), V is the volume and S is the surface area of the virus. The P values shown are for slow and fast quenching events assuming the particle radius is 60 nm and that the enclosed inner volume is “empty”, which likely results in overestimation of the permeability value. Nonetheless, the obtained P values are much lower than those reported in the literature for liposomes. It is thus possible that the viral interior proteins buffer the inner pH by binding the incoming protons and slowing down YFP quenching. (TIF) [file ppat.1011217.s008.tif]

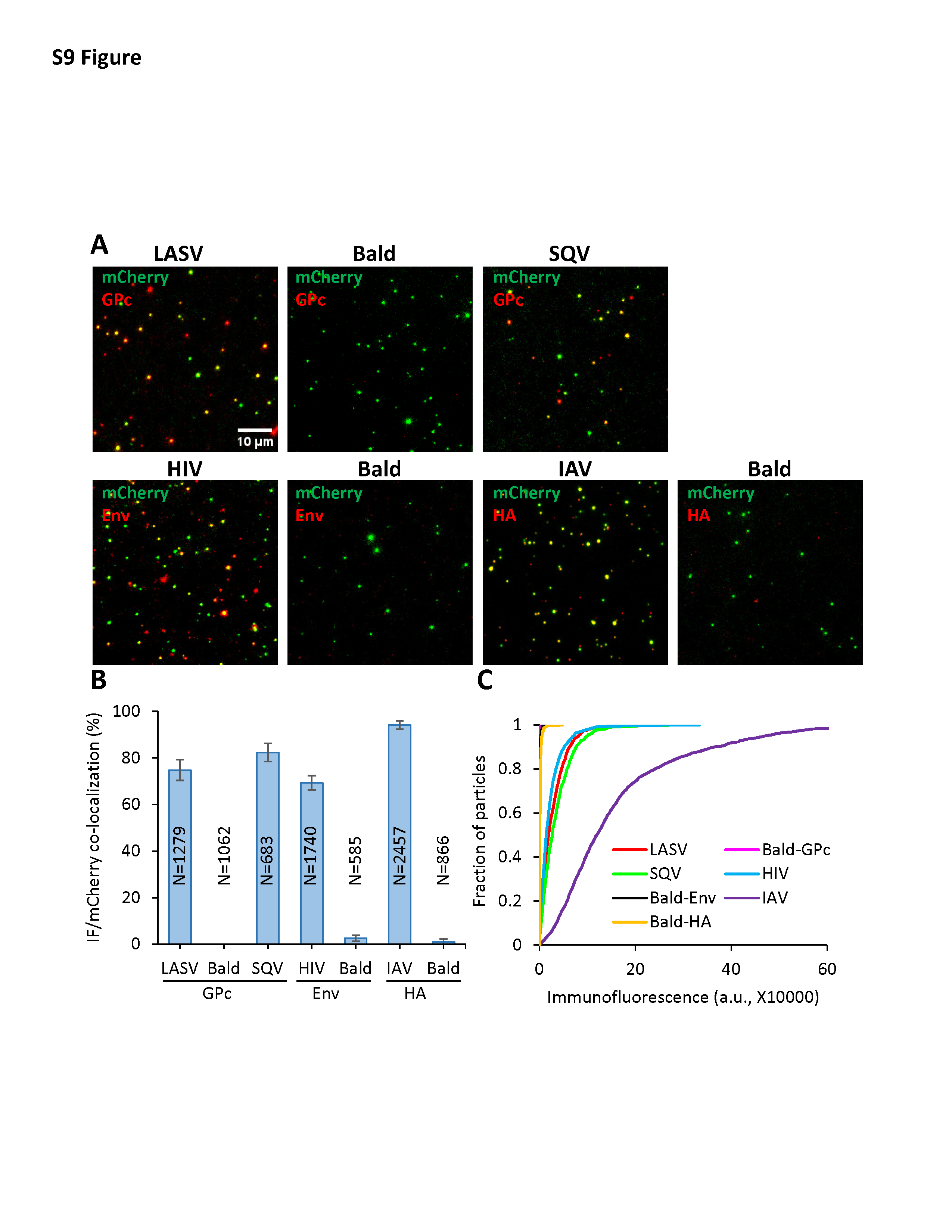

Supplement: S9 Fig — (A) Images of the pseudoviruses labeled with mCherry-CL-YFP-Vpr. Pseudoviruses were bound to poly-L-lysine coated coverslips, fixed, and incubated with anti- LASV-GPc, anti-HIV Env or anti-IAV HA antibodies, as indicated in the figure. Viruses then were stained with respective AF647-conjugated secondary antibodies. Viral particles were identified based on the mCherry signal. Bald particles lacking envelope glycoproteins and GPc-containing viruses produced in the presence of saquinavir viruses were used as controls. (B) Quantification of co-localization of viral glycoproteins detected by immunofluorescence signal and viral particles labeled with mCherry. The numbers of LASVpp analyzed are shown above the bars. Data shown are means ± SD of 4 imaging fields. (C) Distribution of GPc immunofluorescence intensity for different pseudoviruses. (TIF) [file ppat.1011217.s009.tif]

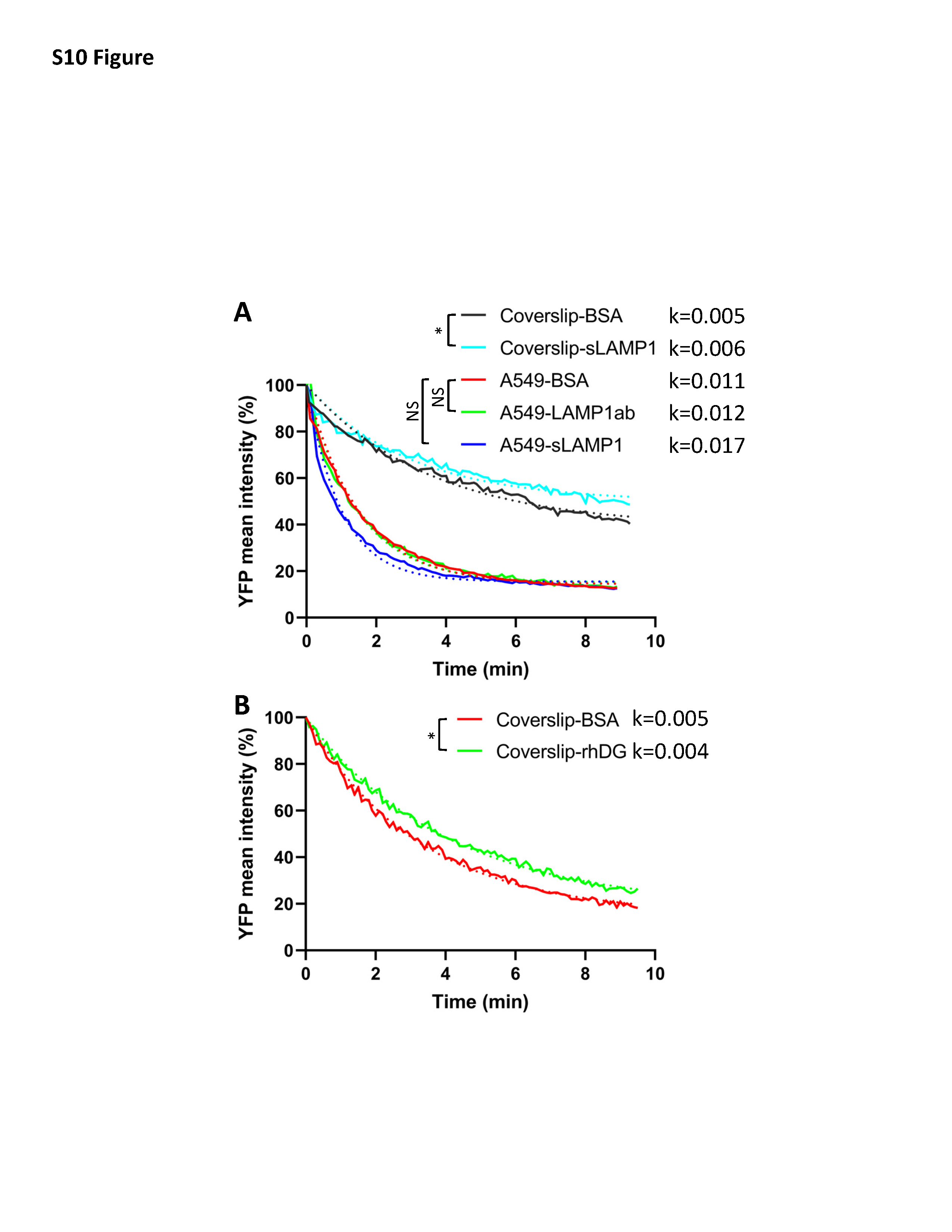

Supplement: S10 Fig — LASVpp were bound to the cell surface or to poly-L-lysine coated coverslips at 4°C. GPc conformational changes are triggered by applying membrane impermeable pH 5.0 citrate buffer. (A) For soluble LAMP1 (sLAMP1) treatment, 200 μg/ml of sLAMP1 was added to viruses in a pH 5.0 citrate buffer. For LAMP1 antibody (LAMP1ab) treatment, cells were incubated with the growth medium containing 100x diluted LAMP1ab for 1 hour before imaging; LAMP1ab was also present in the pH 5.0 citrate buffer. Two hundred μg/ml of BSA was in citrate pH 5.0 buffer used as a control. (B) For recombinant human α-dystroglycan (rhDG) treatment, LASVpp were incubated with 50 μg/ml of rhDG or BSA (control) at 37°C for 20 min, the pH 5.0 citrate buffer was supplemented with 50 μg/ml of BSA or rhDG. The rate constants k are in 1/sec. (TIF) [file ppat.1011217.s010.tif]

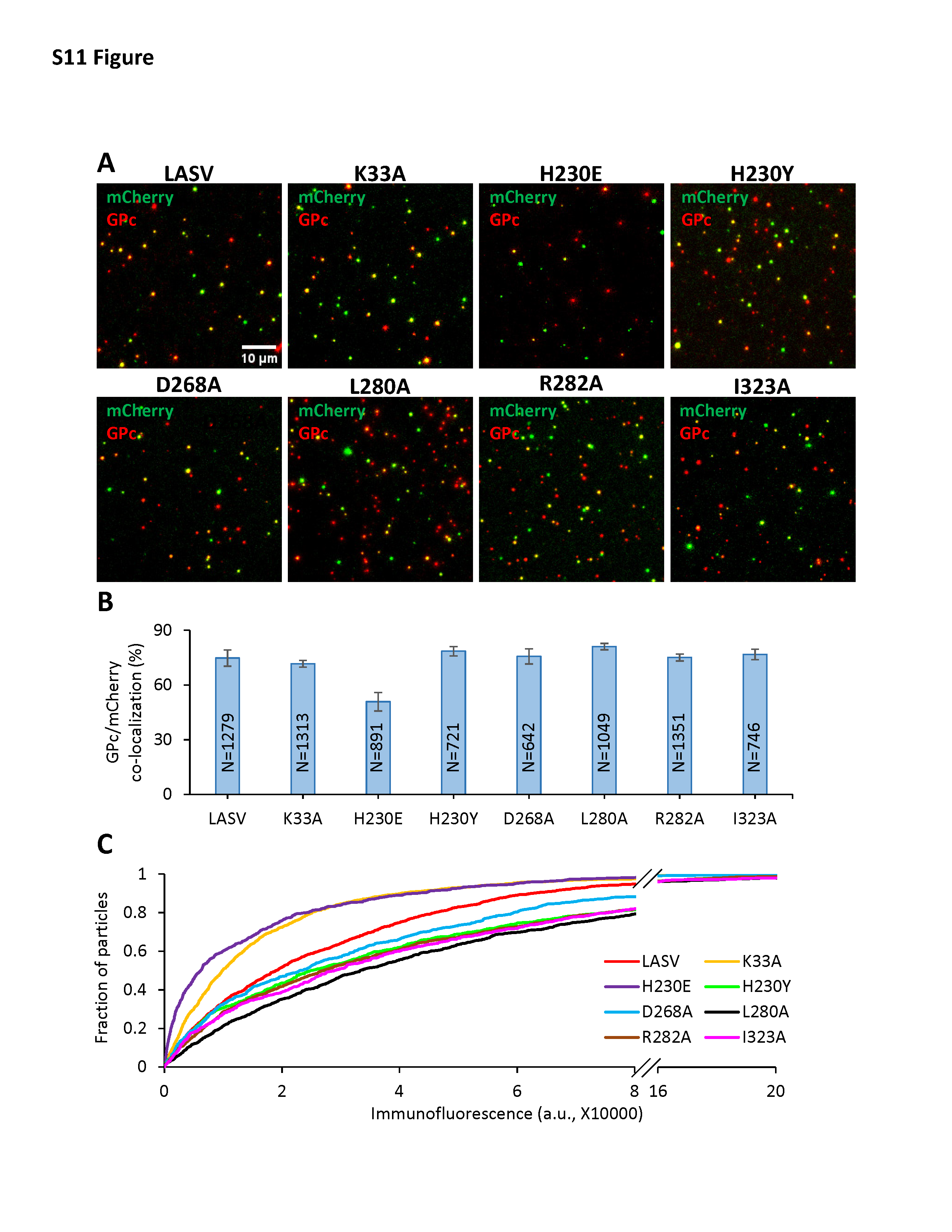

Supplement: S11 Fig — (A) Images of the pseudoviruses labeled with mCherry-CL-YFP-Vpr. Pseudoviruses were bound to poly-L-lysine coated coverslips, fixed, and incubated with anti-LASV GPc human antibody, followed by staining with AF647-conjugated anti-human antibody. Viruses were identified based on the mCherry marker. (B) Quantification of the co-localization of GPc immunofluorescence and viral particles identified by mCherry. The numbers of LASVpp analyzed are shown above the bars. Data shown are means ± SD of 4 imaging fields. (C) Distribution of the GPc immunofluorescence intensities for each virus. (TIF) [file ppat.1011217.s011.tif]
