# Supplementary material for: Fusogenic structural changes in arenavirus glycoproteins are associated with viroporin activity
Source: PLoS Pathog. 2023 Jul 26;19(7):e1011217. doi: 10.1371/journal.ppat.1011217 (PMC10406333; doi:10.1371/journal.ppat.1011217)
Supplement: Attachment — Submitted filename: Arenavirus membrane permeabilization_PPath-rebuttal_4.docx [file ppat.1011217.s017.docx]

**Reviewer #1**:

Much of the data are strong and convincing. Some of the claims are based on the authors’ interpretation of a single event. Further quantification from multiple single tracking instances is needed to validate some of these claims. This single particle tracking method was previously published (Sood et al. 2017) with robust quantification of viral fusion events. The present study would also benefit from the addition of expression levels for the glycoproteins and their mutated versions.

Major points:

1) Figures 1, 2, and 3 rely on single viral fusion instances for their claims. Quantification of multiple instances and showing more expansive views of these interactions, as in movie S3, is needed to validate the author’s claims.

**Response to the general comment and the related point 1**: We agree with the reviewer on this point, and indeed we do not make conclusions based on the few single virus tracking events shown in Fig. 1. Single LASVpp fusion has been extensively characterized in our previous paper (Zhang et al., PLoS Path. 2022), while fusion of LASVpp with different cell types and of MACVpp were shown in the former Fig. S2. These results are moved to the current Fig. 1D, while representative images and traces illustrating single LCMVpp and JUNVpp fusion are moved to the current Fig. S2. We now also include analysis of the kinetics of GPc-driven single pseudovirus fusion across cells lines and different pseudoviruses (Fig. 1E). The results of IAVpp fusion are also included for comparison.

We are perplexed by the notion that Figs. 2 and 3 relied on single fusion instances. These are just representative events out of at least several dozens of events. Analysis pertaining to Fig. 2 was shown in the original Suppl. Fig. S3 (same current figure number), while analysis of the results in Fig. 3 were included in the original submission of that figure.

The mutant GPc incorporation levels were shown in the Suppl. Fig. S11.

2) For the HIV-1 pseudotyped YFP-Vpr studies (Fig. 1), the authors show IAVpp does not contain the leaky virus-endosome phenotype (Suppl. Fig. S1) yet they do not seem to show this necessary control for the arenavirus VLP NP-YFP studies.

**Response:** That is a great suggestion. Unfortunately, we are unable to pseudotype these VLPs with IAV HA. However, we can pseudotype Candid#1 VLPs labeled with NP-YFP/NP-mCherry with VSV G and we now demonstrate the lack of “leaky” single particle fusion (which we now term “pre-fusion viral membrane permeabilization”, PVMP) for VSV-G pseudotyped VLPs (Suppl. Fig. S3A). These VLPs bear the VSV G protein (Fig. S3D, E) and display the expected robust virus-cell fusion activity, as measured by the BlaM assay (Suppl. Fig. S3C)

3) The authors state, “Quantitative differences in the efficiency and kinetics of VLP-cell fusion mediated by different GPcs (Suppl. Fig. S3) are likely caused by varied levels of expression of these glycoproteins or incorporation into Candid-1-based VLPs.”. A western blot or other quantitation of the relative expression levels would help to explain these differences.

**Response:** Per the reviewer’s suggestion, we assessed GPc incorporation into VLPs using a immunofluorescence assay (current Fig. S3D, E). Unfortunately, we were unable to find a pan-arenavirus antibody that would recognize the entire GPC panel and had to use different antibodies for distant GPc species. This does not allow a meaningful comparison of the efficiency of GPc incorporation into VLPs. We therefore stated that we were unable to determine whether the differences in the efficiency and kinetics of VLP fusion reflected differences of GPc incorporation efficiency:

“In the absence of a pan-arenavirus GPc monoclonal antibody, we were unable to directly compare levels of GPc incorporation into the various VLPs and thus could not exclude that such differences may contribute to the observed differences in the extent and kinetics of fusion among the arenavirus VLPs.”

Similarly, the author’s claim, “… the relatively minor fraction of instant YFP quenching events (Fig. 4) does not considerably vary across the mutants…” yet these differences could be attributed to different expression levels in the absence of precise data on expression.

**Response:** We apologize for any confusion caused by the incorrectly numbered figure. With respect to expression levels, these are shown in Supple. Fig 11. Except for H230E mutant, the expression levels of mutant GPc incorporation into virions are similar.

Minor:

1) For Figure 7, GPc mutated protein expression levels would be helpful.

**Response:** We again apologize for the confusion. These levels of mutant GPcs were shown in Suppl. Fig. 11.

2) It would be helpful to merge figures 1 and 2 together so that comparisons between pseudotyped and virus-like particles could be made in the same figure.

**Response:** We utilize the two figures in order to avoid what would be an overly large, confusing and crowded figure. Readers should be able to compare across the two figures.

3) In multiple places percentages are given, without statistical data provided to validate those percentages. For example, it is unclear what is the total number of tracked particles when the author states “… only 14% of cell-bound particles exhibited a loss of pHuji signal within 2 hrs of infection (Fig. 3D) …”.

**Response:** We thank the reviewer for this suggestion and have now incorporated the number of analyzed particles above the figure panels. This also makes explicit the numbers of particles analyzed to arrive at the representative traces.

**Reviewer #2**:

Major points:

The major concern is regarding the extent to which the current results advance our understanding of the role of viral membrane permeabilization, or the stage of entry during which it occurs. Can further efforts be made to specify when during fusion the permeabilization occurs? Is direct interaction between GPc and the endosomal membrane (or plasma membrane) necessary? Based on the discussion in the manuscript, the authors appear to feel that permeabilization occurs prior to GPc interaction with the endosomal membrane. Perhaps evaluation of permeabilization during inhibition of Lassa virus fusion with a heptad repeat-derived peptide would help strengthen this point.

**Response:** We agree that further work is needed to dissect when and how viral membrane permeabilization occurs relative to the fusion process. This study establishes, for the first time, that the pre-fusion viral membrane permeability (PVMP) increases are linked to fusion-associated GPc refolding. We also provide evidence that early conformational changes do not effectively induce permeability increases. We further show that viruses adhered to coverslips and exposed to low pH, in the absence of membrane, do not exhibit an efficient increase in membrane permeability. These results are consistent with PVMP occurring upon GPc interactions with the target membrane.

As to the usage of the GPc heptad-repeat-derived peptides to gain further insights into viral membrane permeabilization, we are not aware of such peptides that can effectively block Lassa entry/fusion (see doi: 10.3389/fmicb.2019.01829 for the lack of inhibition of LASV entry). In fact, a GPc-derived peptide has been shown to activate conformational changes in arenavirus glycoprotein and induce lipid missing with liposomes (doi:10.1128/JVI.01133-14).

In general, it’s difficult to know what to conclude from the data collected on GPc mutants. As the authors note, the K33A mutant is thought to interrupt pH sensing. Does the K33A mutant still have the same dependence on pH for YFP quenching?

**Response:** Based on the marked suppression of “fast” YFP quenching events, we conclude that viral permeability increases are inhibited by an early block of GPc refolding. Per the reviewer’s suggestion, we tested whether exposure to pH <5.0 rescues the ability of the K33A mutant to induce pre-fusion permeabilization of the viral membrane. The new Fig. 6C shows that pH 4.0 and 4.5 treatment of LASVpp-K33A does not majorly increase the rate of YFP quenching relative to pH 5.0 exposure, as compared to WT GPc. These results are fully consistent with the prior finding that no physiological pH can trigger K33A (doi:10.1128/JVI.00642-06).

Furthermore, all the GP1 and GP2 mutants considered reduce fusion by ~90% (or more), and yet have very modest effects, or in some cases no effect, on the kinetics of YFP quenching and the fraction of fast quenching events. How do we interpret the magnitude of these differences in effects of the mutants? The authors argue that permeabilization occurs early and the mutations inhibit fusion at a later step. But ST-193, which inhibits at the point of hemifusion (ie, a late event), has a much larger effect of the kinetics of YFP quenching. How do we reconcile these observations?

**Response:** The GPc mutants were chosen based on their significant defects in fusion. Our interpretation of a modest decrease in the fraction of fast YFP quenching events (Fig. 7) is that these mutations do not fully block GPc refolding into late/final conformation (and thus increased viral membrane permeability) but delayed or inhibited these conformational changes just enough to prevent membrane fusion. Consistently, the GP1 mutations tend to have a stronger effect on permeability increases than the GP2 mutations. The two exceptions to such weak effects of mutations of viral membrane permeability increases are the K33A mutant and WT GPc in the presence of ST-193 inhibitor (Fig. 6). The ST-193 inhibitor is thought to interfere with initiation of GPc refolding at low pH by targeting the interface between the SSP and transmembrane domain of GP2. However, our published results indicate that LASV fusion can proceed as far as hemifusion in the presence of this inhibitor. The fusion arrest at a dead-end hemifusion state is likely a result of impaired/delayed conformational changes of the viral glycoproteins (see, for example, doi: [10.1083/jcb.140.6.1369](https://doi.org/10.1083%2Fjcb.140.6.1369), for the influenza virus hemagglutinin). In Discussion, we speculate that off-path conformational changes in GPc may not effectively promote permeability increases:

“Since suboptimal conditions for triggering viral fusion tend to favor dead-end hemifusion (e.g., [65]), we propose that ST-193 inhibits PVMP, while allowing off-path conformational changes in GPc leading to a non-productive hemifusion state.”

Minor:

The text refers to a L270A mutation in GP2. But Figure 7 refers to L280A.

**Response:** Corrected, thank you.

Figure 7B indicates statistical significance in the rates of YFP quenching for WT, and H230E/Y, and yet the indicated rates are all 0.011.

**Response:** Although the quenching curves and rate constants are nearly identical, the difference between the quenching curves is statistically significant, apparently due to the differences in their plateau levels.

The authors may be interested to note that acidic pH-induced remodeling on the Ebola VP40 matrix due to permeabilization of the viral membrane has also been recently reported (<https://doi.org/10.1101/2022.08.24.505067>).

**Response:** We thank the reviewer for pointing out this manuscript. We now reference the EMBO J. article by this group in Discussion.

**Reviewer #3**: 

The analogy to M2 is worth bringing up in the introduction, since the authors have already shown that LASV has an envelope proton permeability during fusion.

**Response:** We agree that our previous publications support the analogy with M2. We now include the analogy in the Introduction.

Similarly, it is not until the very last paragraph of the paper that all the interesting points are raised, and now it is too dense. I then realize that I cannot tell if a membrane-disrupting fusion peptide can just fall back onto the envelope and cause these changes, but only when on a cell surface. Nor can I tell in WT if there is ever fusion without permeabilization, vs. permeabilization without fusion, because the aggregate data of Fig. 5B is not linked. ST193 is very important, I would suggest the authors devote a paragraph on ST193 alone.

**Response:** We appreciate this comment. It is true that our functional results do not provide structural information regarding the GPc refolding and its link to viral membrane permeabilization. If we must speculate, the body of published work indicates that the fusion peptide preferentially inserts into the viral membrane in the absence of a target membrane. Thus, it does not appear that the viral membrane permeabilization during fusion is directly linked to the fusion peptide insertion.

As to the second point, viral membrane permeabilization without fusion does occur, but it is much less efficient that in fusing particles (Fig. 5).

We are not sure how the results in Fig. 5B should be linked. This graph shows the YFP quenching rate constants categorized by fusing vs. non-fusing particles, which seems to be the link in question.

We have also expanded the discussion of ST-193 results in the Discussion section.

I would have appreciated a better description of what might be expected in the curves of dequenching. There are multiple nonlinear factors that might accelerate the dequenching, yet it is constantly slowing. Perhaps something can be learned by averaging together all the dequenching curves with one condition?

**Response:** YFP dequenching upon fusion is too fast to be resolved in imaging experiments (see Figs. 1 and 2 and Suppl. Fig. S2). This is most likely due to a quick reneutralization of the virus’ interior through a fusion pore connecting it to the cytoplasm. If the reviewer meant gradual YFP quenching, we did show the average YFP quenching profiles in the Fig. 5A.

On page 19, the authors say: “Our results strongly suggest that arenaviral membrane permeabilization is mediated by fusion inducing conformational changes in GPc”. I am not convinced. The experiment shows the difference between on glass and on cell. It may be that cell proteins are involved as well. Perhaps the authors think that “mediated” means “play some role” but I do not read it that way. “it is likely?” perhaps?

**Response:** We thank the reviewer for this comment. Our conclusion is based not solely based upon the difference between coverslip-attached and cell-bound viruses, but also on the marked difference between fusing and non-fusing pseudoviruses in cells (Fig. 5), as well as the effects of fusion-impairing GPc mutations on the membrane permeability increases. In addition, such membrane permeability increases are observed for pseudoviruses and VLPs bearing the arenavirus GPcs and not glycoproteins of other viruses (Figs. 1 and 2 and Suppl. Figs. S1 and S2). We therefore believe that these permeability increases are directly related to fusion inducing GPc refolding and not by host factors. We nonetheless edited that sentence to read:

“Our results strongly suggest that arenaviral membrane permeabilization is related to fusion-inducing conformational changes in GPc.”

Minor:

Stylistic issues:
I do not find the term “leaky fusion” to be very useful anymore. In the old days of viral fusion research, it was very important to distinguish “real” fusion from artefact, and “leaky” fusion was equated with artefact. But the current interest is more on pores in virology, and this is another example of leaks on one side or the other of the hemifusion diaphragm. It seems to be important. To call it “leaky” may do the authors a disservice.

**Response:** We thank the reviewer for this suggestion and have removed “leaky” fusion from the text and figures. To indicate compromised membrane barrier function, we use pre-fusion viral membrane permeabilization (PVMP) or similar terms.

In addition, I find the paper somewhat wordy and stilted, and it obscures the beautiful experiments. I offer one example of my suggestions: Page 13 of the pdf (sorry, the reviewer’s copy did not have page numbers).
a) Cut “…exhibited distinct YFP quenching profiles and…”. Done
b) Shown instead of “manifested”. Done
c) “…particles with presumably preexisiting…”. Edited.
d) “…increase in the baseline viral…” could be “… increased viral…” do you mean increased envelope permeability? What does the baseline have to do with it? To my mind, the baseline is what comes before shifting to pH 4. Edited.

Page 14: middle paragraph could use a conclusion, what is the point of all these numbers?

**Response:** We have added an additional paragraph describing the new data on pH-dependence of viral membrane permeabilization of the K33A GPc mutant (per the Reviewer 2 request) and included conclusions for this section.

Page 15: nine lines from bottom: I do not know enough to understand the link between the last two sentences of this paragraph. Anyway, it seems to be a discussion point, not a result.

**Response:** We apologize for not numbering the lines of the manuscript, which made it difficult for the reviewers to identify the exact paragraphs/sentences that need our attention. If the reviewer refers to the paragraph describing the lack of effects of dystroglycan and soluble LAMP1 on the low pH-induced membrane permeabilization of LASVpp adhered to coverslips, we have edited the text to clarify. We also agree that this is more of a discussion point but prefer to keep the interpretation of these negative results in this paragraph, as this does not appear to constitute a significant point to deserve a separate paragraph in Discussion.

Page 20: last sentence of middle paragraph: I had to put brackets around “…YFP-Vpr quenching” and “pore formation” to get to the 8 min. Perhaps cut “DF-1 cells expressing human LAMP1” or get the 8 min in front?

**Response:** Edited. Thank you.

Finally there is no structural data in this paper, just mutants implying structural changes.

**Response:** We agree. At this stage, getting structural information on GPc intermediates and/or viral membrane defects is not technically feasible.

Figs 1-3 and S1: The cartoon shows “Neutral endosome”. Does such a thing exist? The cartoon makes a big deal out of something but later it seems that it is delayed entry. I would fix this. It is likely that the endosome starts to acidify somewhat early after pinching off. Perhaps the authors could refer to their earlier papers if such a thing exists, and then they would have to add experiments to find the delay between pinching off, acidification, and permeabilization. Perhaps it is simpler to delete this potentially misleading cartoon part?

**Response:** We agree that we cannot state unequivocally if/when acidification starts relative to virion endocytosis. We have removed “neutral” endosomes from the cartoons.

Fig S2: NS or ** compared to which bar? All compared to A549?

**Response:** Sorry for the confusion. All results are compared to LASVpp fusion in A549 cells. We added the description to the figure legends (current Figure. 1D).

Fig S9: Title to legend: “Viral glycoproteins are…” Don’t you want to erase the word “are”?

**Response:** Corrected, thank you.

Movies: I do not see the point of including movies, there are few pixels and the figure data shows the same information. No mention of the movements inside the endosomes are mentioned in the text. I would delete them and place into a database for data sharing. Anyway Movie S3 does not show in mac QuickTime player or in mac Fiji.

**Response:** We apologize for inconvenience. The movies are now uploaded in the MP4 format. We believe that including these movies in the supplement would help the reader to get a better sense of dynamic processes culminating in viral fusion.
